# Integrated toxicology of *Aconitum carmichaelii* Debx.: bridging traditional toxicity-efficacy understanding and modern toxicology

**DOI:** 10.3389/fphar.2025.1667059

**Published:** 2025-11-03

**Authors:** Xin-Yu Li, Li Zhou, Zhen-Hong Jiang, Yong Liang, Ya-Guo Fan, Zhao-Hui Ding, Hao Chen, Wen-Jun Liu, Xiang Zhou, Huan-Hua Xu

**Affiliations:** 1 State Key Laboratory for the Modernization of Classical and Famous Prescriptions of Chinese Medicine, Nanchang, China; 2 Jiang Zhong Pharmaceutical Co., Ltd., Nanchang, China; 3 Key Laboratory of Modern Preparation of TCM, Ministry of Education, Jiangxi University of Chinese Medicine, Nanchang, China; 4 Jiangxi Key Laboratory of Molecular Medicine, The Second Affiliated Hospital of Nanchang University, Nanchang, China; 5 The Affiliated Hospital of Jiangxi University of Chinese Medicine, Nanchang, China

**Keywords:** aconite, integrative toxicology, detoxification, compatibility, pharmacologic effects, toxicology

## Abstract

Toxicity has different meanings in traditional Chinese medicine (TCM) and modern toxicology. Integrative toxicology, a novel discipline proposed by our team, offers a robust solution for the scientific elucidation of toxicity in traditional Chinese medicines. *Aconitum carmichaelii* Debx. (aconite), a classic herbal medicine with a long-standing TCM clinical application history, demonstrates prominent effects in rescuing yang to reverse critical conditions, warming meridians to dispel cold, and tonifying yang to invigorate qi. It is widely used to treat yang deficiency, cold syndromes, and related disorders. However, the dual toxicity-efficacy attribute of aconite has substantially constrained the safety and breadth of its clinical application, leading to its classification as a “high-risk herb.” Thus, this review introduces the concept of integrative toxicology to comprehensively summarize the chemical composition, pharmacological activity, and toxicity mechanisms of aconite. Particular emphasis is placed on various strategies and mechanisms for toxicity attenuation and efficacy enhancement within TCM formulae, including traditional approaches, such as processing and compatibility, as well as potential detoxification pathways identified in modern pharmacological studies. By systematically integrating the framework of integrative toxicology, this work aims to provide a more scientific and secure theoretical basis for the clinical application of aconite, promoting its transformation from a “high-risk herb” to a “controllable therapeutic agent” and thereby maximizing its potential value in modern medicine.

## 1 Introduction

There is a significant difference between the understanding of toxicity in traditional Chinese medicine (TCM) and modern toxicology. TCM regards toxicity as the “bias” of drugs, emphasizes the therapeutic logic of “correcting bias with bias,” and considers toxicity to be a dynamic property of drug effects that should be regulated through individualized administration and compounding (Zhao et al., 2024). In contrast, modern toxicology focuses on the quantitative damage mechanism of exogenous substances to organisms and defines the safety threshold through standardized experimental models with their dose-response relationship as the core. Based on the above differences, our team innovatively proposes to integrate toxicology as an emerging interdisciplinary discipline. Its objective is to analyze the mechanism of toxicity and the law of toxicity reduction using multidisciplinary tools, and to promote the safety evaluation of TCM from “unclear” to “quantifiable and controllable” (Xu et al., 2024).

The processed product of *Aconitum carmichaelii* Debx. (prepared aconite root, the processed lateral root commonly used in TCM) has the effect of warming yang and dispersing cold and returning yang to save the reverse. Clinical applications of *Aconitum carmichaelii* Debx. are seen throughout the history of TCM, especially for yang deficiency and cold condensation and other syndromes, with the best therapeutic effects. It has been listed as the first “emergency medicine” of past generations. However, the *Chinese Pharmacopoeia* has clearly labeled it as “highly toxic,” and the bis-ester-ype alkaloids in aconite are not only the source of cardiotonic and anti-inflammatory pharmacological activity, but also the core causative agents of cardiotoxicity and liver injury (Luo et al., 2018; Gao T. X. et al., 2022). The Pharmacopoeia explicitly stipulates that aconite must be decocted before use, with a dosage range of 3–15 g. The total of diester-diterpenoid alkaloid content must not exceed 0.010% to control the toxicity risk. The lethal dose of pure aconite alkaloid in *Homo sapiens* is 2 mg, 5 mL for aconite tincture, and 1 g for wild plants. This pharmacopeial positioning of “toxicity and efficacy sharing the same origin” makes it difficult to balance toxicity control and efficacy optimization. Traditional research has predominantly focused on single components or isolated mechanisms, making it challenging to systematically elucidate the molecular basis of the dynamic equilibrium between toxicity and efficacy. This highlights the necessity of conducting integrated toxicological research to achieve controllable toxicity (Luo et al., 2018; Ch, 2020; Gao T. X. et al., 2022; Zhang D. X. et al., 2022).

This review is guided by the concept of integrated toxicology and comprehensively synthesizes the chemical composition, pharmacological effects, and toxicity mechanisms of aconite. It focuses on various methods and mechanisms of toxicity reduction and potency enhancement within TCM formulae, such as traditional methods of concoctions and combinations, as well as potential detoxification pathways identified in modern pharmacological studies. By integrating a systematic investigation of toxicology, the aim is to provide a more scientific and safer theoretical basis for the clinical application of aconite, and to promote its transformation from a high-risk herb to a controllable therapeutic agent. This will enable better utilization of its potential value in modern medicine.

## 2 Materials and methods

The present review was conducted using a standardized literature search and screening process to obtain the core data to systematize the toxicity-pharmacological characteristics of aconite (Aconitum carmichaelii Debx.) and integrate the progress of toxicological studies.

### 2.1 Search principles

The literature was searched using the “combination of subject words + free words” search principle, and a parallel search of Chinese and English literature was conducted. We used the subject words to identify core research on the toxicology and pharmacology of aconite and the free words to expand related terms (e.g., “concocting to reduce toxicity” and “compounding to reduce toxicity”), while also considering both traditional medical records and current research advancements.

### 2.2 Databases used

The databases, which included PubMed, Web of Science, CNKI, Wanfang Data, Chinese Pharmacopoeia 2025 Edition, and others, were divided into Chinese databases, English databases, and books.

### 2.3 Timeframe for searching the literature

Although the majority of the cited material was centered between 2017 and 2025, the reference publication dates ranged from 1990 to 2025. Early published literature was searched because the classical theory, the conventional concoction method, *etc.*, are the main subjects of some early research, and have some reference value.

### 2.4 Keywords

The following keywords were searched: “pharmacological effects,” “toxicological effects,” “compounding to reduce toxicity,” “concocting to minimize toxicity,” “aconite,” and some related terms like “Fuzi.”

## 3 Chemical composition studies

Over more than half a century of systematic research, the chemical substance basis of aconite and its processed forms has been fully analyzed. This medicinal plant mainly contains alkaloids, steroids, lipids, organic acids, and trace elements. Among them, some C19-type diesters, such as aconitine, are regarded as the main active ingredients and possess the core pharmacological effects, such as cardiotonic and antitumor effects (Li S. et al., 2019; He et al., 2023). The aconite alkaloids can be categorized into the following four types based on structural characteristics: the diester type (e.g., aconitine, hypaconitine, neoaconitine), the monoester type (e.g., benzoylaconitine), the aminol type (e.g., aconine), and other types (containing structural units such as flavonoids and saponins). The diester-type alkaloids have significant cardiotoxicity, which can be converted to less toxic intermediates through stepwise hydrolysis and metabolism, ultimately resulting in the nearly nontoxic aminol type (Li S. et al., 2019; Hu et al., 2025). In addition, according to the principle of similarity solubility, alkaloids with different alkalinity strengths can be extracted under different pH conditions, such as C19-type diterpene alkaloids are mostly extracted from fat-soluble and weakly alkaline alkaloids, while extracts from strongly alkaline sites are mostly C20-type diterpene alkaloids. The formation of water-soluble alkaloids in aconite is mainly achieved from the alkaline hydrolysis pathways of fat-soluble alkaloids (Xu et al., 2021). The diversity and complexity of these chemical constituents provide a rich material basis for pharmacological studies and clinical applications of aconite.

In addition to alkaloids, flavonoids and polysaccharides in aconite are also involved in its multidimensional pharmacological effects, and their toxicity is significantly less than that of alkaloids. The polysaccharide fraction of aconite is characterized by low toxicity and mild action, with bioactivities mainly including immunomodulation, antitumor, anti-inflammatory, and hypoglycemic effects (Tang et al., 2023; Zhang et al., 2025). Although the flavonoids in aconite are limited in variety, existing pharmacological studies have shown that they have antioxidant and anti-inflammatory activity. However, their specific molecular mechanisms have not been fully elucidated and need to be further investigated (Tang et al., 2017; Fu et al., 2022). The molecular and structural formulas of the alkaloids and their derivatives involved in the aconite are shown in the following table.

## 4 Overview of pharmacological research

**FIGURE 1:**
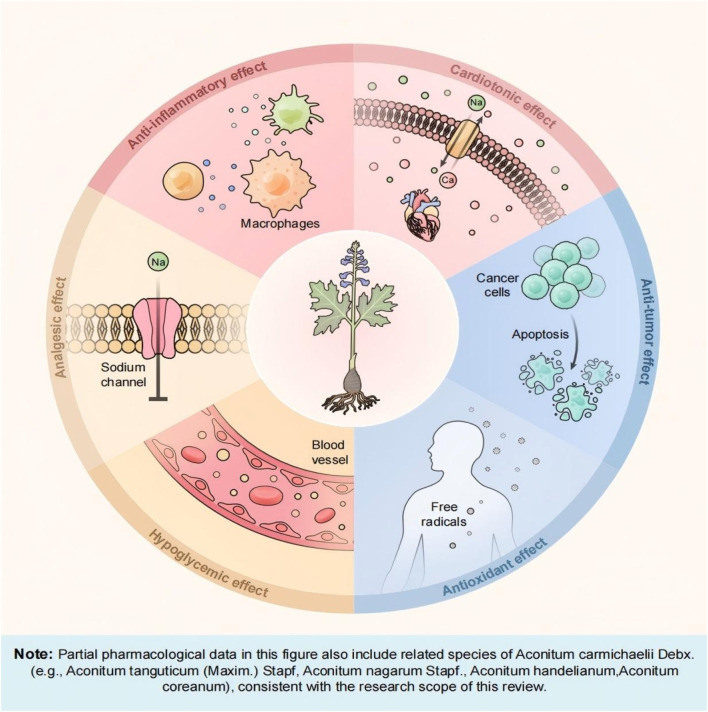
Summary of the main pharmacological effects of aconite.

Chinese medicine plays a leading role in the treatment of many clinical diseases. “Justice of the Materia Medica” recorded aconite “to restore the Yang to save the first product medicine, its power can rise and fall, can reach the internal, can be dispersed.” It can be seen that the aconite can be through the mechanism of bi-directional regulation, to achieve a dynamic balance of the body’s functions, and both the internal internal organs, the external penetration of the surface of the medicinal properties, so that the physiological function of the body systemic regulation and holistic improvement, the chemical components of aconite are the main bearer of multiple pharmacological effects. The following sections summarize the pharmacological effects of aconite and the mechanism of action, as illustrated in Figure 1.

### 4.1 Cardiotonic effects

In Chinese medicine theory, heart failure is attributed to “qi deficiency and yang deficiency,” and aconite, as a TCM that benefits yang, replenishes qi, and restores yang, is still widely used in modern clinical practice for cardiovascular diseases, such as heart failure, with a recommended clinical dosage of 1.5–3 g (Chan et al., 1994; Chan, 2009; Xing et al., 2023). The cardiotonic activity of aconite is mainly attributed to its chemical constituents, including alkaloids, such as demethyl coclaurine and aconitine; glycosides, such as cardiac glycosides; aconite glycosides, and the polysaccharide fraction (Xu et al., 2021; Wang Q. et al., 2023). The mechanism by which aconite exerts its cardiotonic effects can be summarized into the following aspects: (1) Disrupts ion concentrations within myocardial cells: Myocardial cells contain various ions and their corresponding enzymes, which affect cardiac contraction and relaxation. Water-soluble alkaloids increase intracellular Na^+^ concentrations by activating voltage-gated sodium channels, which, in turn, activate the Na^+^/Ca^2+^ exchanger retrograde transport mode and promote Ca^2+^ inward flow to enhance myocardial contractility, while inhibiting calcium-ion overload and apoptosis by regulating Na^+^/K^+^-ATPase activity. *In vitro* investigations showed that the water-soluble alkaloids of aconite at concentrations of 0.02 and 0.04 g/L could decrease the activity of Na^+^-K^+^-ATPase while increasing the activity of Ca^2+^-Mg^2+^-ATPase and Ca^2+^-ATPase on the cell membrane. The myocardial cells of the heart failure model in the treated groups (water-soluble alkaloids) showed noticeably improved cell viability and beating after 1.5 h. When aconitine, an aconite alkaloid, acts on potassium ion channels, it inhibits potassium ion efflux, prolongs the duration of the action potential, and enhances myocardial contractility (He et al., 2014; Xu et al., 2021; Zhou W. et al., 2021). (2) Acts on cardiac receptors: Aconite produces a positive inotropic action by exciting adrenergic receptors, including cardiac β1 and α receptors. The heart rate, cardiac output, and myocardial contractility of rats were all markedly elevated by a 30 g/kg dose of diluted aconite alcohol extract. Additionally, cardiac cell damage was decreased, and the TCM had a cardiotonic impact by inhibiting the overexpression of cytokines, such as nitric oxide (NO), tumor necrosis factor (TNF-α), and serum interleukin-6 (IL-6) (Zhao et al., 2012; Xing et al., 2022; Yang Y. et al., 2022). (3) Interferes with signaling pathways: The active ingredients in aconite can also regulate related proteins and signaling pathways, such as modulating apoptosis-associated proteins, protecting myocardial cells, and inhibiting cell apoptosis. Animal experimental results demonstrated that aconite decoction administered at doses of 5 g/kg, 2.5 g/kg, and 1.25 g/kg could significantly upregulate the PI3K/Akt signaling pathway (Wang et al., 2019; Liu et al., 2025).

The polysaccharide fraction of aconite also shows significant pharmacological activity in improving cardiac function, which can scavenge reactive oxygen species (ROS) and reduce mitochondrial damage, thus slowing cardiomyocyte apoptosis and minimizing cardiomyocyte damage. It also regulates the autonomic nervous system, enhances sympathetic nerve activity, and produces positive inotropic effects to further enhance myocardial contractility (Zhang J. et al., 2022).

### 4.2 Anti-inflammatory and immunomodulatory effects

Aconite has remarkable anti-inflammatory and immunomodulatory activity, which is mainly mediated by its diverse chemical components, especially the fat-soluble alkaloids. The anti-inflammatory mechanism of aconite (*Aconitum tanguticum (*Maxim.) Stapf, a related species of *Aconitum carmichaelii* Debx.) is primarily related to effects to inhibit pro-inflammatory signaling pathways and reduce cytokine release, such as alleviating inflammatory damage by blocking the nuclear factor (NF)-κB and MAPK signaling pathways and acting on macrophages (Ye et al., 2021; Ye et al., 2023). Among them, the mechanisms involving alkaloids primarily encompass the following aspects: (1) Benzoylaconine (BAC) exerts its effects by inhibiting the expression of Toll-like receptor 4 (TLR-4), reducing the phosphorylation of upstream TAK1, and subsequently blocking the activation of downstream NF-κB and MAPK pathways. For example, in cell experiments, RAW 264.7 macrophages activated by lipopolysaccharide (LPS) were treated with BAC at concentrations of 1 μM, 10 μM, and 100 μM. At all three concentrations, BAC inhibited the production of pro-inflammatory cytokines (IL-6, TNF-α, and IL-1β) and inflammatory mediators (NO), while downregulating the expression of iNOS and COX-2, thereby exerting anti-inflammatory effects (Zhou C. et al., 2021). (2) In contrast, the water-soluble component demethyl coclaurine exerts its effects by inhibiting the NF-κB pathway and activating the Nrf2/HO-1 signaling axis. For example, when 0.5 μmol/L of demethyl *Lindera aggregata* alkaloid was applied to BV2 cells under inflammatory conditions (LPS activation) for 1 day, it significantly downregulated the expression of inflammatory markers, such as TNF-α, IL-6, NO, and prostaglandin E2 (Yang et al., 2020; Xu et al., 2021). (3) Aconite can also exert anti-inflammatory effects by downregulating costimulatory molecules, such as CD80, to inhibit dendritic cell maturation, while balancing Th1/Th2 differentiation and blocking the inflammatory cascade response. At a concentration of 2 mg/mL, formulations containing *Aconitum carmichaelii* significantly decreased the overexpression of IL-1β, IL-12, interferon (IFN)-γ, and IL-6 (Ji et al., 2017).

In addition to alkaloids, the polysaccharide fraction of aconite also plays a key role in immune regulation, with effects characterized by low toxicity and mild bioactivity. Its immune regulatory effects are manifested in multiple aspects: (1) Studies have shown that the polysaccharide fraction of aconite can enhance the organ coefficients of the spleen and thymus, promote the proliferation of splenic lymphocytes and abdominal macrophages, and increase the serum concentrations of NO and IFN-γ in mice. (2) The polysaccharides in Heshun tablets can antagonize cyclophosphamide-induced immunosuppression by activating immune effector cells, such as macrophages, and regulate immune homeostasis through both intrinsic and adaptive immunity. The polysaccharide fraction of aconite extract can also inhibit the differentiation of macrophages to pro-inflammatory phenotypes and alleviate excessive inflammatory responses (Hu Q. et al., 2023; Li et al., 2023). (3) Aconite polysaccharides can also improve immune function by regulating the composition of intestinal flora, elevating short-chain fatty acid levels and reversing decreases in the immune organ index and the abnormal expression of inflammatory factors in immunosuppressed mice (Ran T. et al., 2023).

### 4.3 Antitumor effects

In the basic theory of TCM, the pathogenesis of cancer can be summarized as “deficiency of yang qi, deficiency of the positive and the negative, and deficiency of the basic and the standard.” Aconite, as a representative herb for warming the yang and dispersing the cold, with its unique mechanism of “supporting the positive and consolidating the basic and warming the yang qi,” has demonstrated important value as an adjuvant treatment for tumors (He et al., 2024). Pharmacological studies have shown that the antitumor effects of aconite itself and its compound preparations are related to the alkaloidal components it contains and work well. Its anticancer mechanisms primarily include regulating signaling pathways, activating pro-apoptotic factors, modulating gene-protein pathways, and enhancing immune functions. These effects are achieved through the following four dimensions: (1) Diester alkaloids from aconite can inhibit tumor cell proliferation by regulating multiple signaling pathways and suppressing the expression of cell cycle-related proteins. For example, in melanoma, aconitine exerts antitumor effects by downregulating the MAPK/ERK1/2 and PI3K/AKT signaling pathways, thereby reducing the expression of the cell cycle-related protein proliferating cell nuclear antigen. Additionally, when liver cancer cells were treated with aconitine at concentrations below 20 μg/mL, alkaloids significantly inhibited the activation of the P38/ MAPK signaling pathway, thereby suppressing liver cancer cell proliferation (Du et al., 2013; Xiong et al., 2018; Gao Y. B. et al., 2022; Zhang W. et al., 2022). (2) The various alkaloids in aconite also exhibit varying degrees of regulatory effects on pro-apoptotic factors, manifested as inducing cancer cell apoptosis, reducing tumor volume, activating the mitochondrial-dependent apoptotic pathway, and exerting antitumor effects by enhancing autophagy. For example, aconitine at concentrations of 15–60 μM can significantly upregulate the pro-apoptotic factor Bax, inhibit the proliferation of pancreatic carcinoma cells, and induce apoptosis. In animal experiments, a dose of 100 mg/kg of aconitine significantly suppressed tumor growth and induced apoptosis (Ji et al., 2016; Zhang W. et al., 2022). (3) Network pharmacological studies have shown that the antitumor effects of aconite are also related to its regulation of the adenosine phosphorylase gene and related protein pathways (Lu et al., 2021). (4) Aconite water extract can also serve as an immune adjuvant by activating c-Jun N-terminal kinase to enhance the infiltration level of natural killer cells, thereby strengthening immune function and achieving antitumor effects (Yang et al., 2023).

In addition to alkaloids, phenolic compounds and the polysaccharide fraction in aconite also exhibit significant antitumor activity. Their mechanism of action involves upregulating the expression levels of pro-apoptotic proteins, such as Bax, regulating apoptotic pathways, mediating the process of apoptosis in tumor cells, inhibiting the abnormal proliferation of tumor cells, and blocking the key pathway of tumor angiogenesis (Zhang W. et al., 2022).

### 4.4 Analgesic effects

Pharmacological studies have confirmed that the alkaloids contained in aconite are of great value in treating mild-to-moderate pain, such as neuropathic pain, osteoarthralgia, and cancer pain, by modulating the nerve conduction pathway and inhibiting the release of inflammatory mediators (Luo et al., 2020; Li L. et al., 2022). In four classic mouse pain models, the hot plate model, the acetic acid-induced writhing phantom model, and the administration of aconitine at concentrations of 0.3 mg/kg and 0.9 mg/kg demonstrated significant antinociceptive activity, exhibiting marked therapeutic effects on acute thermal stimulation pain, visceral pain, and inflammatory pain (Deng et al., 2021). The mechanism of its analgesic effect can be summarized into the following aspects: (1) Overall analgesic mechanism: Diterpenoid alkaloids of aconite (*Aconitum nagarum* Stapf*,* a related species of *Aconitum carmichaelii* Debx.), as the central components of the analgesic mechanism, act mainly through a voltage-gated sodium channel blockade mechanism (Hu J. et al., 2023; Zhang W. et al., 2024). (2) Central analgesic mechanism: Aconite can activate spinal microglial cells and stimulate β-adrenergic receptors to exert analgesic effects. A microinjection (5–100 ng/rat) of *Aconitum carmichaelii* alkaloids into specific intracranial nuclei increased central norepinephrine levels within 30 min of administration, and accelerated norepinephrine turnover rates in the brainstem and spinal cord (Sun et al., 2020; Li S. L. et al., 2022). (3) Peripheral analgesic mechanism: Aconitine acts directly on dorsal root ganglion neurons in the non-central nervous system to reduce the generation and conduction of pain. The active component neoline extracted from the processed aconitum polysaccharide fraction was reported to significantly inhibit peripheral neural pain, such as cold sensation, pain sensation, and mechanical pain, in mice when subcutaneously injected at a concentration of 10 mg/kg for 4 days (Suzuki et al., 2016; Xu et al., 2021). In addition, analgesic effects are also achieved by activating the G protein/PI3K/PIP2 signaling pathway and shutting down TRPV1 channels (Xiao et al., 2019; Xu et al., 2021; Gao Y. B. et al., 2022).

### 4.5 Other pharmacological effects

In addition to the aforementioned pharmacological activities, aconite also exhibits various biological activities, such as antioxidant, hypoglycemic, and anti-arrhythmia effects. Research on the active components and their mechanisms of action provides experimental evidence for expanding clinical applications.

#### 4.5.1 Antioxidant effects

Studies have shown that the antioxidant effects of C19-type diterpenoid alkaloids contained in *Aconitum handelianum* H.F.Comber (a synonym of *Aconitum pulchellum* var. *pulchellum* Hand.-Mazz.) mainly depend on active groups, such as phenolic hydroxyl and ammonia groups, in the molecular structure (Yin et al., 2016).

#### 4.5.2 Hypoglycemic effects

The polysaccharides from aconite (*Aconitum carmichaelii* Debx. and *Aconitum coreanum*) exhibit significant activity in glucose metabolism regulation, particularly in improving insulin resistance and glycemic homeostasis. This is achieved by promoting peripheral tissue glucose utilization, enhancing insulin sensitivity, and regulating lipid metabolism, ultimately leading to glycemic homeostasis regulation. The anti-inflammatory RG-II-type polysaccharide (KMPS) purified from *Aconitum coreanum* decreases the serine phosphorylation of insulin receptor substrates in the liver, alleviates inflammation in serum and insulin target tissues, and improves glucose metabolic disorders. After 4 weeks of KMPS treatment at 400 mg/kg, serum insulin and C-peptide levels were reduced in diet-induced obese mice, along with significant decreases in free-fatty acid and triglyceride levels (Su et al., 2020; Fu et al., 2022; Zhang et al., 2025).

#### 4.5.3 Anti-arrhythmia effects

Unlike the single active component-dominant mode observed to produce antioxidant and hypoglycemic effects, the antiarrhythmia action of aconite exhibits multi-target characteristics. Both its alcohol extract and water extract demonstrate significant inhibitory effects on ventricular fibrillation, primarily mediated by characteristic C18- and C19-type diterpenoid alkaloids. The specific mechanisms include C18-type diterpenoid alkaloids (e.g., lappaconitine), targeting the regulation of cardiomyocyte Na^+^ channels. At doses of 0.05–0.15 mg/kg, these diterpenoid alkaloids can suppress the occurrence of premature ventricular beats and ventricular tachycardia. C19-type diterpenoid alkaloids regulate the pathological process of arrhythmia by inhibiting myocardial oxidative stress, modulating mitochondrial energy metabolism homeostasis, and interacting with key biomolecules (Liu et al., 2023).

Although the mechanisms of the above three pharmacological effects are different, they jointly reflect the diversity of the chemical components of aconite and their ability to systematically regulate the physiological functions of the body, providing more possibilities for its application in the treatment of complex diseases.

## 5 Overview of toxicological studies

### 5.1 Cardiotoxicity

The cardiotoxicity of aconite (*Aconitum leucostomum* Worosch, a related species of *Aconitum carmichaelii* Debx.) possesses the most prominent toxicological characteristic, with the primary toxic components being diester C19-diterpenoid alkaloids. *In vitro* toxicity experiments in the rat H9c2 cardiomyocyte cell line showed a half-maximal inhibitory concentration (IC50) of aconitine of 562.06 μg/mL and significant dose-dependency. Other C19-type alkaloid components, such as delvestidine and anthranoyllycoctonine, exhibited markedly higher toxicity compared to aconitine (Nie et al., 2017). The mechanisms of cardiotoxicity induced by aconite can be roughly summarized as follows: (1) Electrophysiological mechanisms: Aconitine triggers persistent sodium inward flow by inhibiting the inactivation of cardiomyocyte voltage-gated sodium channels (Nav1.5), leading to the abnormal depolarization of cardiomyocyte membrane potentials (Coulson et al., 2017; Zhang X. C. et al., 2020). (2) Calcium homeostasis mechanism: The sustained opening of Na^+^ channels leads to the persistent opening of L-type calcium channels, thereby further triggering intracellular Ca^2+^ homeostasis imbalance manifested as tachyarrhythmia. Animal experiments demonstrated that 1 μmol/L *Aconitum carmichaelii* alkaloid promoted Ca^2+^ influx in rats, while 5 and 10 μmol/L induced ventricular arrhythmia in rat cardiomyocytes (Zhou et al., 2013; Jiang et al., 2021). (3) Molecular targets: Mechanistic studies have demonstrated that alkaloids from *Aconitum carmichaelii* disrupt myocardial calcium signaling and electrophysiological balance, ultimately inducing arrhythmia through pathways including interfering with sarcoplasmic reticulum ryanodine receptor (RyR2) function, upregulating RyR_2_ expression, enhancing sarcoplasmic reticulum calcium release, and inhibiting L-type calcium channels (Fu et al., 2008; Chen R. C. et al., 2013). (4) Mitochondrial dysfunction: Mitochondrial dysfunction plays a significant role in the cardiotoxicity of *Aconitum carmichaelii* alkaloids. These alkaloids can induce mitochondrial oxidative stress and impair ATP synthesis, mediating cardiomyocyte apoptosis and lipid peroxidation. H9c2 cardiomyocytes treated with 25 g/L of aconite water extract for 1 day showed increased mitochondrial ROS levels, decreased mitochondrial membrane potential, and evident mitochondrial damage (Zhao et al., 2015; Jiang et al., 2021). (5) Clinical manifestations: In clinical practice, aconite poisoning often presents as multiple coexisting features caused by intersecting mechanisms. Symptoms can manifest in as little as 10 min and include palpitation, chest distress, tachycardia, and, in severe cases, the effects may even progress to heart failure (Lin et al., 2004; Sun et al., 2018; Hao et al., 2020).

### 5.2 Hepatotoxicity

Although the heart is the main target organ of aconite toxicity, its hepatotoxic effects have gradually attracted academic attention. Toxicokinetic analysis revealed that the active ingredient of aconite showed multi-organ distribution after absorption, in which the concentration of liver tissue distribution was significantly higher than that of other organs (Hao et al., 2020). Hepatotoxicity can be identified by the following: (1) Animal toxicology experiments: Male Wistar rats were continuously gavaged with the water extract of Hei shunpian (HSP, processed aconite root) for 20 days. Serum transaminase levels were significantly elevated in both the low- dose HSP group (20 g/kg) and the high-dose HSP group (40 g/kg), with an accumulation of lipid peroxidation products in liver tissues (Zhang K. et al., 2020). (2) Histopathological observation: Mesaconine can cause histopathological changes in rat liver tissue at specific dose thresholds. After a single oral mesaconine gavage dose of 10 mL/kg to Sprague-Dawley (SD) rats, followed by 4 h of fasting and continuous observation for 2 weeks, the liver of rats in each administration group exhibited fatty vacuoles or degeneration, along with hepatocyte necrosis (Chen et al., 2023). (3) Molecular mechanisms: The regulation of targets, such as RAC-alpha serine/ threonine-protein kinase 1 (AKT1), interleukin-2 (IL2), coagulation factor II (F2, also known as prothrombin), glutathione reductase (GSR), and epidermal growth factor receptor (EGFR), affects pathways including T-helper 17 cell (Th17 cell) differentiation, the Janus kinase-signal transducer and activator of transcription (Jak-STAT) signaling pathway, and glutathione metabolism, inducing oxidative stress, metabolic disorders, cell apoptosis, immune responses, and the excessive release of inflammatory factors, ultimately leading to liver injury. HepG2 cells treated with aconitine demonstrated that the uptake of multiple diester- type alkaloids may rely on organic cation/proton antiport transporters, thereby achieving distribution in the liver (Cong et al., 2019; Zhang K. et al., 2020).

### 5.3 Acute toxicity

(1) Clinical and pathological characteristics: The acute toxicity of aconite is characterized by multi-organ dysfunction syndrome, with clinical symptoms including behavioral inhibition (idleness and prone stillness), gastrointestinal reactions (nausea), motor nerve disorders (limb paralysis), and central nervous system excitation-inhibition imbalance (paroxysmal muscle tonus and convulsions), with significant dose-dependent pathological changes. In an aconitine toxicity experiment in SD rats, the low-dose administration groups (1.00 and 2.15 mg/kg) exhibited toxic characteristic responses, such as lethargy and spasms, within 1 day, but returned to normal after 1 day. In contrast, the medium- and high-dose administration groups (4.64 and 10.0 mg/kg) showed toxic characteristics within 2 h and died successively within 4 h (Li et al., 2013; Chen et al., 2023). (2) Core toxicological mechanisms: Aconitine inhibits voltage-gated sodium channel inactivation, triggering the persistent sodium current-mediated depolarization of neuronal and myocardial cell membrane potentials, leading to fatal ventricular arrhythmia. This mechanism can also induce a series of toxic reactions, such as neuromuscular transmission blockade (reduced acetylcholine release). One patient ingested approximately 120 g of steamed aconite slices and developed palpitations and generalized numbness within 1 h, subsequently falling into a coma. Additionally, three patients died from severe systemic damage after consuming over 50 g of aconite (Yang X. et al., 2017; Chen et al., 2023). (3) Factors affecting toxicity: It is noteworthy that the toxicokinetic differences of *Aconitum carmichaelii* after administration may be closely related to cytochrome P450 enzyme polymorphism and processing parameters. Rat microsomes were selected for the *in vitro* analysis of aconitine metabolism measured by high-performance liquid chromatography (HPLC). The results indicated that CYP3A4 was responsible for the primary metabolism of aconitine. Moreover, rats of different sexes exhibited varying tolerance levels to the acute oral toxicity of aconitine (Ye et al., 2011; Chen et al., 2023). (4) Prevention strategies: Traditional water decoction and high-pressure moist heat processing techniques can effectively degrade the toxic components of diterpenoid alkaloids, significantly reducing the probability of acute poisoning. For example, the water extract of processed black aconite slices (a derivative of raw aconite) at doses of 0.8 g/kg, 1.6 g/kg, and 3.2 g/kg showed no effects on rat body weight compared to the raw aconite water extract group, while cardiovascular indicators, such as cardiac index exhibited a decrease (Chen P. et al., 2013; Li et al., 2024).

### 5.4 Other toxicity

In addition to the aforementioned toxic reactions, the toxic effects of aconitine also involve multi-organ damage to the nervous system, digestive system, and other organs.

#### 5.4.1 Neurotoxicity

(1) Clinical manifestations: Based on multiple adverse clinical cases of aconitum, the clinical manifestations of neurotoxicity mainly include paresthesia, tremors, and the disturbance of consciousness, which can be summarized by four key characteristics: ① Numbness (manifested as limb numbness, tongue numbness, and other features); ② Tremors (manifested as convulsions, muscle rigidity); ③ Confusion (manifested as speech disorders, dizziness, and blurred consciousness); ④ Exhaustion (manifested as dyspnea and weakness) (Yang X. et al., 2017). (2) Toxicity mechanisms: ① Aconitine was shown to persistently activate voltage-gated sodium channels and inhibit Na^+^-K^+^-ATPase activity, leading to abnormal intracellular Na^+^-ATPase activity, which triggers an imbalance in the homeostasis of intracellular ions, such as Na^+^, K^+^, and Ca^2+^. This process progresses to disrupt both the central and peripheral nervous systems, resulting in symptoms like numbness and tremors. *Aconitum carmichaelii alkaloids* induced the lipid peroxidation of the cell membrane of interstitial cells of Cajal (ICC), damaging nerve cells (Peng et al., 2009). ② *Aconitum carmichaelii* alkaloids blocked signal transmission at the neuromuscular junction and inhibited neurotransmitter release from the presynaptic membrane in mice. The dose-dependent inhibition of neurally evoked twitch tension in the diaphragm at concentrations of 0.3–2 μM, while no effect was seen on contractions induced by direct muscle stimulation. This experimental background validates the aforementioned mechanism (Muroi et al., 1990). ③ Directly damages neuronal cells to exert neurotoxicity: Extracts from three different species of *Aconitum carmichaelii* (Radix aconiti, Radix *Aconiti Kusnezoffii*, Radix *Aconiti Lateralis Praeparata*) all exhibit toxic effects on hippocampal neuronal cells, inhibiting their growth and survival (Han et al., 2007).

#### 5.4.2 Digestive system toxicity

(1) Clinical manifestations of digestive system toxicity: The digestive system toxicity manifestations based on the clinical characteristics of multiple cases of aconite adverse reactions include nausea and vomiting and abdominal pain (Yang X. et al., 2017). (2) Toxicological mechanism: It is speculated that the mechanism may be related to the regulation of intestinal nerve ion channels (e.g., Ca+) and interference with the contraction rhythm of gastrointestinal smooth muscle cells by aconitine. In guinea pig experiments, aconitine stimulated the release of acetylcholine from postganglionic cholinergic nerves, inducing strong ileal contractions and thereby causing diarrhea, abdominal pain, and other symptoms (Lin et al., 2004; Chan, 2009).

## 6 Overview of detoxification research

### 6.1 Processes reducing toxicity

As the earliest surviving heirloom text that systematically records the concoction technology of aconite, Jin Gui Yu Han Jing laid the theoretical foundation for the control of toxic components in TCM. In TCM, the concoction process of aconite is of key significance in reducing its inherent toxicity and enhancing the safety of clinical use. The mechanism of aconite preparations to reduce toxicity has experienced a systematic historical evolution and technological innovation from traditional pretreatment processes to the classical method to modern innovative technology (Chen R. C. et al., 2013; Hu et al., 2025). The aconite pretreatment methods include peeling, breaking, and raw use. The peeling of Chinese medicine is aimed at purifying the drug and facilitating concoctions and clinical use of the drug. After aconite has been peeled to remove its root skin, its toxic components are significantly reduced, and alkaloids can be easily solubilized. “Breaking open” refers to increasing the heat area of the herbs through physical division and destroying toxic substances, such as biester alkaloids, after prolonged high-temperature decoction, thus realizing the purpose of reducing toxicity and increasing efficacy. Although raw aconite retains its inherent toxicity, it has the unique effect of quickly breaking yin and dispersing cold (Hou et al., 2024). The concoctions of aconite include fire, brine, steam, and boiling methods. The diversified concoctions of aconite provide multiple paths for its safe clinical application and the optimization of medicinal efficacy (Dong et al., 2020).

**FIGURE 2:**
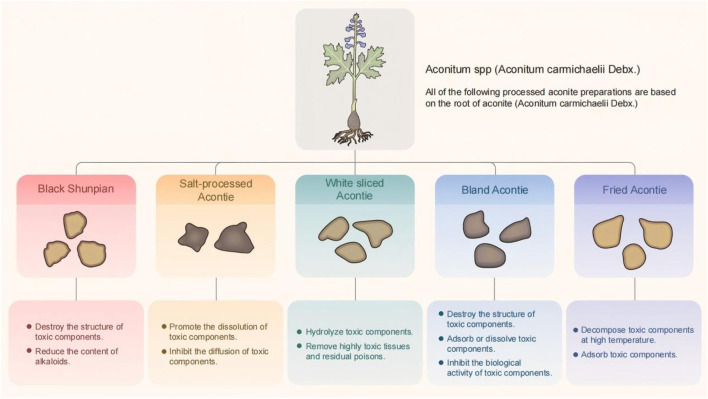
The five types of aconite concoctions are summarized according to the mechanism of toxicity reduction.

The *Chinese Pharmacopoeia* records five types of processed Radix *Aconiti Lateralis Praeparata* (processed forms of *Aconitum carmichaelii* Debx., commonly referred to as prepared aconite root), whose detoxification mechanisms are all directly related to the chemical degradation of diester-diterpenoid alkaloids (e.g., aconitine and hypaconitine) into monoester-diterpenoid alkaloids (e.g., benzoylaconine and benzoylhypaconine). The degradation pathways include the cleavage of the C-8 ester bond of diester-diterpenoid alkaloids under high-temperature steam to form monoester-diterpenoid alkaloids, whose toxicity is 1/200 to 1/500 that of diester diterpenoid alkaloids. As hydrolysis conditions intensify, the C-14 ester bond breaks, forming amino alcohol-type aconitine, whose toxicity is 1/2000 to 1/4000 that of diester-type aconitine (He, 2022): (1) Black shunpian: Mud aconite is washed and dipped in gall bladder water for a few days, boiled until it is heated through the heart, water-bleached and sliced, dipped and bleached to adjust the color, baked to half-dry after steaming, and then finally, dried in the sun or dried. After processing, the structure of biester-type alkaloids in aconite is destroyed and hydrolyzed into the low-toxicity mono- ester-type alkaloids. After processing by boiling for 8 min, water soaking and rinsing four times within 24 h, steaming for 3 h, and drying at 60 °C for 7.5 h, the hypaconitine and aconitine content in processed *Aconitum carmichaelii* slices fell below the detection limit. Additionally, the total content of diester-diterpenoid alkaloids significantly decreased after steaming, while the content of monoester-diterpenoid alkaloids notably increased, likely due to the conversion of diester-diterpenoid alkaloids during the steaming process (Ch, 2020; Gong et al., 2022). (2) Salt-processed aconite: Large and uniform aconite is washed, immersed in gall bladder water overnight and then soaked in salt and sun-dried daily until there is a large amount of salt cream on the surface and the texture becomes hard. This concocting process decreases the total alkaloid content in aconite to achieve the purpose of reducing the toxicity; however, the efficacy of aconite is also decreased after salt processing (Ch, 2020; Peng et al., 2022). (3) White sliced aconite: Uniform-sized mud aconites are soaked in brine for several days, boiled until thoroughly cooked, then peeled and longitudinally sliced. After water soaking, steaming, and sun-drying processes, HPLC testing showed that although the diester-type alkaloid content in the peeled aconite was reduced to approximately one-eighth the amount in raw aconite, it remained significantly higher than in other processed products. The white sliced aconite processing method involves removing the epidermis and high-temperature boiling, which decreases the diester-type alkaloid content and increases monoester-type alkaloid levels. Additionally, 5-hydroxymethylfurfural, a distinctive component not found in other processed forms, emerges, likely due to the high- temperature hydrolysis of *Vitis vinifera* sugars or fructose after epidermal removal (Zhu et al., 2011; Ch, 2020; Zhang C. et al., 2024). (4) Prepared aconite lateral root slices (with glycyrrhiza and black beans): Salt-processed aconite is soaked in clean water to remove salt, then boiled with *Glycyrrhiza uralensis* (common name: licorice root) and black beans until numbing sensations and bitterness disappear. *Glycyrrhiza uralensis* and the black beans are removed, then the aconite is sliced thinly and sun-dried. High-temperature steaming destroys the toxic components, while excipients help adsorb and promote the dissolution of toxic constituents. After processing with black beans and *Glycyrrhiza uralensis*, HPLC analysis shows increased monoester-diterpenoid alkaloid content and decreased diester-diterpenoid alkaloid levels (Guo et al., 2015; Ch, 2020; Yu et al., 2023). (5) Processed aconite lateral root slices: Black or white aconite lateral root slices are used as raw materials and sand-fried until puffed and slightly discolored. High-temperature processing reduces the diester-diterpenoid alkaloid content, thereby decreasing the leaching of toxic components. No diester-diterpenoid alkaloids (e.g., aconitine and neoaconitine) were detected in the processed slices. However, the monoester-diterpenoid alkaloid content decreased to 47.2, 58.1, and 67.1% of that in black aconite lateral root slices, respectively, with the total amount of the three monoester-diterpenoid alkaloids reduced to 54.9% of the raw product (Peng et al., 2019; Ch, 2020). The five detoxification mechanisms of processed aconite are summarized in Figure 2.

In recent years, significant research progress has been made on the detoxification mechanism of aconite by integrating traditional processing techniques and modern technologies. In contemporary aconite processing methods, the pressurized steaming technique effectively degrades diester-type alkaloids while preserving the cardiotonic activity of monoester-type alkaloids by applying high pressure at approximately 120 °C, achieving a detoxification rate exceeding 90%. When processed under moist heat and pressure at 120 °C for 1–1.5 h, the diester-type alkaloids in aconite fall below the detection limit, while the average detected content of monoester-type alkaloids was 1.567 mg/g (Tang et al., 2013). Microwave processing accelerates the decomposition of toxic components by regulating water molecule movement. Under the combined action of magnetic fields and microwaves, water molecules generate substantial heat, hydrolyzing diester-type alkaloids in raw aconite slices, thereby achieving efficient and rapid detoxification (He, 2022).

### 6.2 Detoxification by compatibility

Junchen Zuoshi is the core theory of Chinese medicine prescriptions. In compound formulae, the king’s medicine dominates the therapeutic effect, and the adjuvants assist the king and minister’s efficacy through synergistic enhancement, toxicity control, slowing, and reporting mechanisms (Chen and Tan, 2020). As an important part of the diagnosis and treatment system of TCM, scientific compounding can effectively reduce the toxic side effects and produce synergistic effects. As a representative toxic herb, aconite holds significant value in classical formula research. Its detoxification mechanisms acting through compatibility include forming complexes by precipitating aconitine, interfering with aconitine metabolism, regulating ion channels, and modulating physiological microenvironments. This section will systematically review classic compatibility combinations of aconite (such as *Glycyrrhiza uralensis*-aconite), analyze their compatibility mechanisms, and summarize classical formulas containing aconite along with their clinical applications.

#### 6.2.1 *Glycyrrhiza uralensis* combined with aconite

The combination of *Glycyrrhiza uralensis* and Aconite is commonly used in an herbal pair with classic formulations such as Gancao Fuzi Decoction and Sini Decoction. *Glycyrrhiza uralensis* contains triterpenoid saponins, like glycyrrhizic acid, and flavonoid active components, such as liquiritin, which mitigate the toxicity of aconite through various physical or chemical mechanisms (Wang X. X. et al., 2023; Dang et al., 2024). The common compatibility detoxification mechanisms are as follows: (1) Formation of complexes: Triterpenoid saponin components from *Glycyrrhiza uralensis* can reduce the content of diester-type alkaloids such as aconitine. The hydrolysis product, glucuronic acid from *Vitis vinifera*, combines with aconitine to form nontoxic complexes that are excreted through urine (Chen and Xu, 2006). The flavonoids in *Glycyrrhiza uralensis* (e.g., glycyrrhizin and liquiritin) can precipitate with the diester-diterpenoid alkaloids in aconite, reducing the leaching of toxic components and slowing the absorption of alkaloids in the intestines. After decocting *Glycyrrhiza uralensis* with aconite, the dissolution rate of *Glycyrrhiza uralensis* flavonoids decreases, likely due to the binding of hydroxyl groups in the flavonoids with the diester-diterpenoid alkaloids (Yang et al., 2003; Li Q. P. et al., 2018; Li W. et al., 2018). (2) Metabolic interference: The components of *Glycyrrhiza uralensis* induce the hepatic drug-metabolizing enzyme CYP3A4, elevate the expression levels of CYP3A4-related proteins, enhance enzymatic activity, and accelerate metabolism, thereby reducing peak plasma drug concentrations to achieve detoxification. When the cocktail probe drug method was employed to analyze the metabolism of aconitine in liver microsomes, the results demonstrated a significant acceleration in the metabolic rate of diester-type alkaloids, such as aconitine (Miao et al., 2014; Feng et al., 2024). (3) Regulation of ion channels: Aconitine exhibits significant cardiotoxicity by disrupting intracellular ion homeostasis, causing the sustained influx of Na^+^ and Ca^2+^. In contrast, the glycyrrhizin compounds in *Glycyrrhiza uralensis* can counteract the cardiotoxicity of aconite by acting on Na^+^ channels. For example, glycyrrhetinic acid at concentrations of 0.1, 1.0, and 10.0 μmol L^-1^ can act on L-type calcium channels in myocardial cells, inhibiting Ca^2+^ influx to exert detoxification effects (Xie et al., 2005). Moreover, when 2 mg/kg of glycyrrhiza uralensis flavonoids was administered to rats, they could antagonize the ventricular arrhythmia induced by aconitine (Ma et al., 2019).

#### 6.2.2 *Panax ginseng* combined with aconite

*Panax ginseng* C.A. Mey. (common name: ginseng) is commonly used in TCM to restore yang and boost qi. The most representative formulas in the combined application of aconite and ginseng include Shenfu Decoction and Huiyang Jiuji Decoction. The common detoxification mechanisms are as follows: (1) Regulation of ion channels: *P. ginseng* saponin Rg1 inhibits the influx of Na^+^, K^+^, and Ca^+^ by acting on the ion channels of myocardial cells, thereby reducing their content in myocardial cells. This mechanism antagonizes cardiotoxicity, such as arrhythmia induced by *Aconitum carmichaelii* and mitigates myocardial cell damage (Xu et al., 2022). (2) Metabolic Interference: The combination of *P. ginseng* and aconite can also act on hepatic CYP450 enzymes to achieve detoxification effects. It accelerates the hydroxylation response during the metabolic process of aconite by upregulating the expression of CYP1A2 and CYP3A1 mRNA, thereby reducing cumulative toxicity. After the continuous intragastric administration of aconite water extract alone for 8 days, pathological sections of rat liver tissue showed significant congestion, hepatocyte necrosis, and inflammation. In contrast, pathological sections of liver tissue from rats administered with a 1:1 water extract of *P. ginseng* and aconite appeared normal, with significantly higher expression of CYP1A2 and CYP3A1 mRNA and protein compared to the group treated with aconite alone (Li H. et al., 2019). (3) Chemical degradation: The combination of *P. ginseng* and aconite can also promote the hydrolysis of diester alkaloids in aconite into monoester alkaloids, reducing the content of toxic components. Fatty acids in *P. ginseng* undergo nucleophilic substitution reactions to convert diester-type alkaloids (e.g., aconitine) into monoester-type alkaloids (e.g., benzoylaconine). After the co-decoction of *P. ginseng* and aconite, the content of aconitine decreased by 45% compared to aconite decoction alone, while the content of monoester-type alkaloids increased significantly (Ma et al., 2011; Bao et al., 2022; Qiu et al., 2022).

#### 6.2.3 Compatibility of aconite with other Chinese herbal medicines

In addition to ginseng and licorice, the combination of aconite with other herbs can significantly reduce toxicity. The common formulas include Ganjiang Fuzi Decoction (combining *Zingiber officinale* Rosc. (common name: dried ginger) with aconite), Zhenwu Decoction (*Paeonia lactiflora* Pall. (common name: white peony root), *Atractylodes macrocephala* Koidz. (common name: large-head atractylodes rhizome), *Poria cocos* (Schw.) Wolf (common name: poria), and aconite) and Dahuang Fuzi decoction (comprising *Rheum palmatum* L. (common name: rhubarb), *Asarum sieboldii* Miq. (common name: fine-leaf as arum), and aconite). The detoxification mechanisms can be summarized as follows: (1) Chemical degradation: The carboxyl-containing acidic components in dried ginger undergo acid-base neutralization with aconitine, promoting the hydrolysis of diester-type alkaloids into lipid-formaldehyde-type alkaloids. This process reduces the diester-type alkaloid content and consequently decreases toxicity (Yue et al., 2007; Liu et al., 2024). (2) Formation of complexes: White peony root paeonol in glycosides has weak acid properties and forms ionic pairs with diester alkaloids, which promotes the distribution of monoester alkaloids (such as benzoyl aconitine) and reduces toxicity (Yang H. S. et al., 2017). The tannins in rhubarb combined with aconite form an insoluble complex, which hinders the absorption of diester alkaloids in the digestive system, delaying its intestinal absorption and decreasing the peak blood drug concentration, playing a detoxifying role (Chen et al., 2022).

**FIGURE 3:**
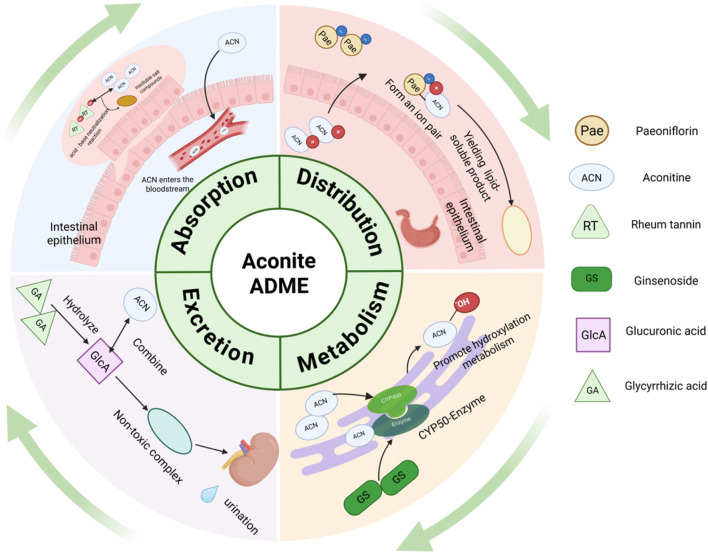
Pharmacokinetic mechanisms of aconitine combined with other herbs for reducing the toxicity of aconite (Created in BioRender (2025) https://BioRender.com/kvhhz6q).

The combinations of other Chinese medicinal materials and aconite are summarized in Table 2 below. The combination of aconite and Chinese medicinal materials can interfere with the absorption, distribution, metabolism, and excretion of toxic aconitine components in the body to achieve the effect of reducing toxicity (Figure 3).

## 7 Conclusion

Aconite (*Aconitum carmichaelii* Debx.) is a cornerstone herb in TCM used for restoring yang energy and dispelling cold. It demonstrates multifunctional benefits, including cardiotonic effects, anti-inflammatory properties, and anticancer activity. Clinically, it is used to treat conditions such as heart failure, rheumatoid arthritis, and myocardial infarction. However, the C19 diester alkaloid compound, its pharmacologically active component, also serves as the root cause of its toxicity. Aconite primarily exhibits cardiac toxicity, hepatotoxicity, and acute toxicity. This stark contradiction between efficacy and toxicity has made reducing its harmful effects while preserving therapeutic benefits a central challenge in advancing its clinical application. Current research indicates that aconite’s toxicity management has evolved through a traditional-modern collaborative approach from ancient processing methods like skin removal and fire-breaking in the *Treatise on Cold Pathogenic Diseases,* to modern microwave detoxification techniques.

Integrated toxicology, leveraging its multidimensional and multi-omics technological advantages, provides a novel approach for systematically deciphering the correlation between aconite’s toxic effects and efficacy while establishing scientific frameworks for toxicity control and functional optimization. Guided by this concept, this study systematically reviewed the chemical composition (Table 1), pharmacological actions (Figure 1), toxic effects, and detoxification mechanisms of aconite (Figure 2; Table 2). This review reveals a persistent core challenge in current research: although the toxicity mechanisms of aconite were validated through various experimental methods, including animal studies, cell experiments, and microsomal system analyses, the transition between the pharmacodynamic effects and toxicological properties of diester alkaloids remains unclear, making it difficult to establish precise dose-response relationships.

**TABLE 1:** Chemical structures and molecular formula of the alkaloids in the aconite and its derivatives.

Formula name	Molecular formula	Chemical structure
Aconitine	C_34_H_47_NO_11_	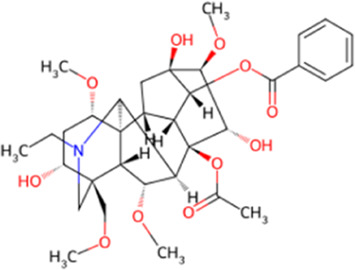
Benzoylaconitine	C_32_H_45_NO_10_	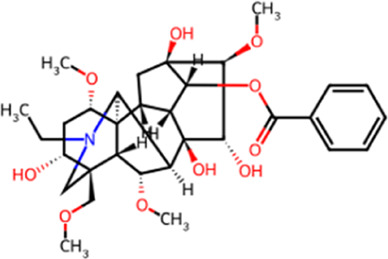
Aconine	C_25_H_41_NO_9_	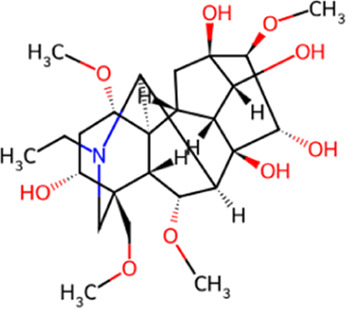
Hypaconitine	C_33_H_45_NO_10_	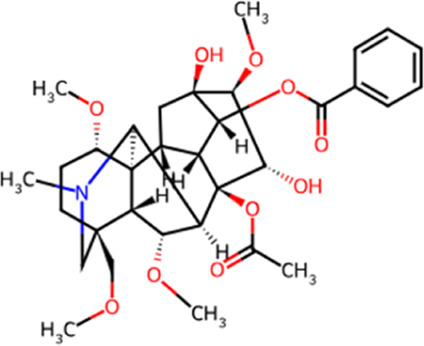
Neoaconitine	C_33_H_45_NO_11_	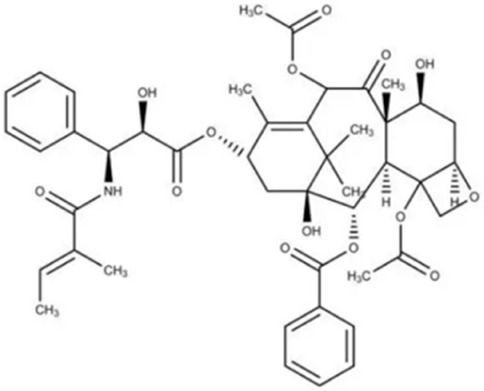

**TABLE 2:** Summary of the compositions and modern clinical applications of aconites in aconite-containing formulae.

Formula name	Compounded herbs	Modern clinical application	References
Fuzi Decoction	*Panax ginseng*, *Atractylodes macrocephala*, *Poria cocos*, *Paeonia lactiflora*	Rheumatoid arthritis	Qin et al. (2022)
Sini Decoction	*Zingiber officinale* *Glycyrrhiza uralensis*	Myocardial infarction, chronic heart failure	Li and Yu (2012), Hong et al. (2020)
Mahuang Fuzi Xixin Decoction	*Ephedra sinica, Asarum sieboldii*	Viral influenza, asthma, allergic rhinitis	Li and Yu (2012), Zhang et al. (2023)
Huiyang Emergency Decoction	*Zingiber officinale, Cinnamomum cassia, Panax ginseng*	Acute gastroenteritis, chronic heart failure	Li and Yu (2012), Zhang et al. (2024b)
Shenfu Decoction	*Panax ginseng*	Chronic heart failure, myocardial infarction, cerebral ischemia	Li and Yu (2012), Lu et al. (2025)
Fuzi Lizhong Decoction	*Codonopsis pilosula* (Franch.) *Atractylodes macrocephala, Zingiber officinale, Glycyrrhiza uralensis*	Chronic gastritis	Hong et al. (2020), Ran et al. (2023a)
Gancao Fuzi Decoction	*Glycyrrhiza uralensis*	Rheumatoid arthritis, osteoarthritis	(Zhang et al., 2022a)
Dahuang Fuzi Decoction	*Rheum palmatum, Asarum sieboldii*	Appendicitis, acute pancreatitis, intestinal obstruction	Hong et al. (2020), Yang et al. (2022a)
Yiyi Fuzi Baijiang Powder	*Coix lacryma-jobi* L.var*. mayuen* (Roman.) Patrinia scabiosaefolia Fisch. ex Trev	Pelvic inflammation, ulcerative colitis	Wei et al. (2024)
Guizhi Fuzi Decoction	*Cinnamomum cassia*	Rheumatoid arthritis, gouty arthritis	Jiang et al. (2024)
Ganjiang Fuzi Decoction	*Zingiber officinale*	Chronic renal failure	Mu and Yu (2024)
Fuzi Xiexin Decoction	*Rheum palmatum, Coptis chinensis, Scutellaria baicalensis*	Chronic atrophic gastritis, chronic renal failure	Shi et al. (2025)
Zhen Wu Decoction	*Atractylodes macrocephala, Poria cocos, Zingiber officinale, Paeonia lactiflora*	Nephrotic syndrome, chronic glomerulonephritis	Chen et al. (2025)
Wen Pi Decoction	*Rheum palmatum, Angelica sinensis* (Oliv.), *Zingiber officinale, Natrii sulfas, Panax ginseng*, *Glycyrrhiza uralensis*	Acute and chronic renal insufficiency, chronic renal failure	Zhang and Liu (2010)

In future research, we will build on the concept of integrated toxicology to further clarify the “efficotoxic boundary” between the active and toxic components in aconite. This will help establish regulatory mechanisms for other aconite constituents in modulating their therapeutic effects. By integrating traditional detoxification expertise with modern technologies, we aim to advance similar dual-toxicity Chinese herbs like *Pinellia ternata, Polygonum multiflorum*, and Asarum. These efforts will enable the maximization of TCM’s clinical value while ensuring enhanced safety and controllability.
