# Correction: Integrated toxicology of *Aconitum carmichaelii* Debx.: bridging traditional toxicity-efficacy understanding and modern toxicology

**DOI:** 10.3389/fphar.2026.1841383

**Published:** 2026-05-12

**Authors:** Xin-Yu Li, Li Zhou, Zhen-Hong Jiang, Yong Liang, Ya-Guo Fan, Zhao-Hui Ding, Hao Chen, Wen-Jun Liu, Xiang Zhou, Huan-Hua Xu

**Affiliations:** 1 State Key Laboratory for the Modernization of Classical and Famous Prescriptions of Chinese Medicine, Nanchang, China; 2 Jiang Zhong Pharmaceutical Co., Ltd., Nanchang, China; 3 Key Laboratory of Modern Preparation of TCM, Ministry of Education, Jiangxi University of Chinese Medicine, Nanchang, China; 4 Jiangxi Key Laboratory of Molecular Medicine, The Second Affiliated Hospital of Nanchang University, Nanchang, China; 5 The Affiliated Hospital of Jiangxi University of Chinese Medicine, Nanchang, China

**Keywords:** aconite, integrative toxicology, detoxification, compatibility, pharmacologic effects, toxicology

The term “synergistically” was incorrectly used.

A correction has been made to the Section 4 Overview of pharmacological research, *4.3 Antitumor effects*, Paragraph number 1:

“In the basic theory of TCM, the pathogenesis of cancer can be summarized as “deficiency of yang qi, deficiency of the positive and the negative, and deficiency of the basic and the standard.” Aconite, as a representative herb for warming the yang and dispersing the cold, with its unique mechanism of “supporting the positive and consolidating the basic and warming the yang qi,” has demonstrated important value as an adjuvant treatment for tumors (He et al., 2024). Pharmacological studies have shown that the antitumor effects of aconite itself and its compound preparations are related to the alkaloidal components it contains and work well. Its anticancer mechanisms primarily include regulating signaling pathways, activating pro-apoptotic factors, modulating gene-protein pathways, and enhancing immune functions. These effects are achieved through the following four dimensions: (1) Diester alkaloids from aconite can inhibit tumor cell proliferation by regulating multiple signaling pathways and suppressing the expression of cell cycle-related proteins. For example, in melanoma, aconitine exerts antitumor effects by downregulating the MAPK/ERK1/2 and PI3K/AKT signaling pathways, thereby reducing the expression of the cell cycle-related protein proliferating cell nuclear antigen. Additionally, when liver cancer cells were treated with aconitine at concentrations below 20 μg/mL, alkaloids significantly inhibited the activation of the P38/MAPK signaling pathway, thereby suppressing liver cancer cell proliferation (Du et al., 2013; Xiong et al., 2018; Gao Y. B. et al., 2022; Zhang W. et al., 2022). (2) The various alkaloids in aconite also exhibit varying degrees of regulatory effects on pro-apoptotic factors, manifested as inducing cancer cell apoptosis, reducing tumor volume, activating the mitochondrial-dependent apoptotic pathway, and exerting antitumor effects by enhancing autophagy. For example, aconitine at concentrations of 15–60 μM can significantly upregulate the pro-apoptotic factor Bax, inhibit the proliferation of pancreatic carcinoma cells, and induce apoptosis. In animal experiments, a dose of 100 mg/kg of aconitine significantly suppressed tumor growth and induced apoptosis (Ji et al., 2016; Zhang W. et al., 2022). (3) Network pharmacological studies have shown that the antitumor effects of aconite are also related to its regulation of the adenosine phosphorylase gene and related protein pathways (Lu et al., 2021). (4) Aconite water extract can also serve as an immune adjuvant by activating c-Jun N-terminal kinase to enhance the infiltration level of natural killer cells, thereby strengthening immune function and achieving antitumor effects (Yang et al., 2023).”

Incorrect and irrelevant plant terminology were used. Specifically, *Aconitum handelianuma*, was related to *Aconitum carmichaelii* Debx., rather than *Aconitum pulchellum* var. *pulchellum* Hand.-Mazz. Furthermore, the taxonomy was not presented in full for *Aconitum handelianum* H.F.Comber. Furthermore, mentions of *Broussonetia papyrifera* has been removed.

A correction has been made to the section 4 Overview of pharmacological research, *4.5 Other pharmacological effects*, 4.5.1 Antioxidant effects, Paragraph Number 1: “Studies have shown that the antioxidant effects of C19-type diterpenoid alkaloids contained in *Aconitum handelianum* H.F.Comber (a synonym of *Aconitum pulchellum* var. *pulchellum* Hand.-Mazz.) mainly depend on active groups, such as phenolic hydroxyl and ammonia groups, in the molecular structure (Yin et al., 2016).”

There were typographical errors as well as an irrelevant plant study included. For example, ‘antiarrhythmia’ was incorrectly given as ‘anti-arrhythmia’, while ‘synergistic’ was incorrectly used alongside ‘characteristics’. Furthermore, mentions of *Parazacco spilurus* subsp. *spilurus* has been removed.

A correction has been made to the section 4 Overview of pharmacological research, *4.5* Other *pharmacological effects*, 4.5.3 Anti-arrhythmia effects, Paragraph Number 1: “Unlike the single active component-dominant mode observed to produce antioxidant and hypoglycemic effects, the antiarrhythmia action of aconite exhibits multi-target characteristics. Both its alcohol extract and water extract demonstrate significant inhibitory effects on ventricular fibrillation, primarily mediated by characteristic C18- and C19-type diterpenoid alkaloids. The specific mechanisms include C18-type diterpenoid alkaloids (e.g., lappaconitine), targeting the regulation of cardiomyocyte Na+ channels. At doses of 0.05–0.15 mg/kg, these diterpenoid alkaloids can suppress the occurrence of premature ventricular beats and ventricular tachycardia. C19-type diterpenoid alkaloids regulate the pathological process of arrhythmia by inhibiting myocardial oxidative stress, modulating mitochondrial energy metabolism homeostasis, and interacting with key biomolecules (Liu et al., 2023).”

Irrelevant plant terminology was used. For example, mentions of ‘*Parazacco spilurus* subsp*. spilurus*’ has been removed in point (2).

A correction has been made to the section 5 Overview of toxicological studies, *5.1 Cardiotoxicity*, Paragraph Number 1: “The cardiotoxicity of aconite (*Aconitum leucostomum* Worosch, a related species of *Aconitum carmichaelii* Debx.) possesses the most prominent toxicological characteristic, with the primary toxic components being diester C19-diterpenoid alkaloids. *In vitro* toxicity experiments in the rat H9c2 cardiomyocyte cell line showed a half-maximal inhibitory concentration (IC_50_) of aconitine of 562.06 μg/mL and significant dose-dependency. Other C19-type alkaloid components, such as delvestidine and anthranoyllycoctonine, exhibited markedly higher toxicity compared to aconitine (Nie et al., 2017). The mechanisms of cardiotoxicity induced by aconite can be roughly summarized as follows: (1) Electrophysiological mechanisms: Aconitine triggers persistent sodium inward flow by inhibiting the inactivation of cardiomyocyte voltage-gated sodium channels (Nav1.5), leading to the abnormal depolarization of cardiomyocyte membrane potentials (Coulson et al., 2017; Zhang X. C. et al., 2020). (2) Calcium homeostasis mechanism: The sustained opening of Na^+^ channels leads to the persistent opening of L-type calcium channels, thereby further triggering intracellular Ca^2+^ homeostasis imbalance manifested as tachyarrhythmia. Animal experiments demonstrated that 1 μmol/L *Aconitum carmichaelii* alkaloid promoted Ca^2+^ influx in rats, while 5 and 10 μmol/L induced ventricular arrhythmia in rat cardiomyocytes (Zhou et al., 2013; Jiang et al., 2021). (3) Molecular targets: Mechanistic studies have demonstrated that alkaloids from *Aconitum carmichaelii* disrupt myocardial calcium signaling and electrophysiological balance, ultimately inducing arrhythmia through pathways including interfering with sarcoplasmic reticulum ryanodine receptor (RyR2) function, upregulating RyR_2_ expression, enhancing sarcoplasmic reticulum calcium release, and inhibiting L-type calcium channels (Fu et al., 2008; Chen R. C. et al., 2013). (4) Mitochondrial dysfunction: Mitochondrial dysfunction plays a significant role in the cardiotoxicity of *Aconitum carmichaelii* alkaloids. These alkaloids can induce mitochondrial oxidative stress and impair ATP synthesis, mediating cardiomyocyte apoptosis and lipid peroxidation. H9c2 cardiomyocytes treated with 25 g/L of aconite water extract for 1 day showed increased mitochondrial ROS levels, decreased mitochondrial membrane potential, and evident mitochondrial damage (Zhao et al., 2015; Jiang et al., 2021). (5) Clinical manifestations: In clinical practice, aconite poisoning often presents as multiple coexisting features caused by intersecting mechanisms. Symptoms can manifest in as little as 10 min and include palpitation, chest distress, tachycardia, and, in severe cases, the effects may even progress to heart failure (Lin et al., 2004; Sun et al., 2018; Hao et al., 2020).”

Inappropriate wording was used, for example, ‘fatty vacuoles’ was incorrectly presented as ‘fatty utetheisa kong vacuoles’.

A correction has been made to the Section 5 Overview of toxicological studies, *5.2 Hepatotoxicity*, Paragraph Number 1: “Although the heart is the main target organ of aconite toxicity, its hepatotoxic effects have gradually attracted academic attention. Toxicokinetic analysis revealed that the active ingredient of aconite showed multi-organ distribution after absorption, in which the concentration of liver tissue distribution was significantly higher than that of other organs (Hao et al., 2020). Hepatotoxicity can be identified by the following: (1) Animal toxicology experiments: Male Wistar rats were continuously gavaged with the water extract of Hei shunpian (HSP, processed aconite root) for 20 days. Serum transaminase levels were significantly elevated in both the low-dose HSP group (20 g/kg) and the high-dose HSP group (40 g/kg), with an accumulation of lipid peroxidation products in liver tissues (Zhang K. et al., 2020). (2) Histopathological observation: Mesaconine can cause histopathological changes in rat liver tissue at specific dose thresholds. After a single oral mesaconine gavage dose of 10 mL/kg to Sprague-Dawley (SD) rats, followed by 4 h of fasting and continuous observation for 2 weeks, the liver of rats in each administration group exhibited fatty vacuoles or degeneration, along with hepatocyte necrosis (Chen et al., 2023). (3) Molecular mechanisms: The regulation of targets, such as RAC-alpha serine/threonine-protein kinase 1 (AKT1), interleukin-2 (IL2), coagulation factor II (F2, also known as prothrombin), glutathione reductase (GSR), and epidermal growth factor receptor (EGFR), affects pathways including T-helper 17 cell (Th17 cell) differentiation, the Janus kinase-signal transducer and activator of transcription (Jak-STAT) signaling pathway, and glutathione metabolism, inducing oxidative stress, metabolic disorders, cell apoptosis, immune responses, and the excessive release of inflammatory factors, ultimately leading to liver injury. HepG2 cells treated with aconitine demonstrated that the uptake of multiple diester-type alkaloids may rely on organic cation/proton antiport transporters, thereby achieving distribution in the liver (Cong et al., 2019; Zhang K. et al., 2020).”

Irrelevant plant terminology was used. For example, mentions of ‘*P. spilurus* subsp*. spilurus*.‘ has been removed in point (2).

A correction has been made to the Section 5 Overview of toxicological studies, *5.4 Other toxicity*, 5.4.1 Neurotoxicity, Paragraph Number 1: “(1) Clinical manifestations: Based on multiple adverse clinical cases of aconitum, the clinical manifestations of neurotoxicity mainly include paresthesia, tremors, and the disturbance of consciousness, which can be summarized by four key characteristics: ① Numbness (manifested as limb numbness, tongue numbness, and other features); ② Tremors (manifested as convulsions, muscle rigidity); ③ Confusion (manifested as speech disorders, dizziness, and blurred consciousness); ④ Exhaustion (manifested as dyspnea and weakness) (Yang X. et al., 2017). (2) Toxicity mechanisms: ① Aconitine was shown to persistently activate voltage-gated sodium channels and inhibit Na^+^-K^+^-ATPase activity, leading to abnormal intracellular Na^+^-ATPase activity, which triggers an imbalance in the homeostasis of intracellular ions, such as Na^+^, K^+^, and Ca^2+^. This process progresses to disrupt both the central and peripheral nervous systems, resulting in symptoms like numbness and tremors. *Aconitum carmichaelii alkaloids* induced the lipid peroxidation of the cell membrane of interstitial cells of Cajal (ICC), damaging nerve cells (Peng et al., 2009). ② *Aconitum carmichaelii* alkaloids blocked signal transmission at the neuromuscular junction and inhibited neurotransmitter release from the presynaptic membrane in mice. The dose-dependent inhibition of neurally evoked twitch tension in the diaphragm at concentrations of 0.3–2 μM, while no effect was seen on contractions induced by direct muscle stimulation. This experimental background validates the aforementioned mechanism (Muroi et al., 1990). ③ Directly damages neuronal cells to exert neurotoxicity: Extracts from three different species of *Aconitum carmichaelii* (Radix aconiti, Radix *Aconiti Kusnezoffii*, Radix *Aconiti Lateralis Praeparata*) all exhibit toxic effects on hippocampal neuronal cells, inhibiting their growth and survival (Han et al., 2007).”

Irrelevant plant terminology was used. For example, mentions of ‘*Broussonetia papyrifera* ‘has been removed in point (4).

A correction has been made to the section 6 Overview of detoxification research, *6.1 Processes reducing toxicity*, Paragraph number 2: “The *Chinese Pharmacopoeia* records five types of processed Radix *Aconiti Lateralis Praeparata* (processed forms of *Aconitum carmichaelii* Debx., commonly referred to as prepared aconite root), whose detoxification mechanisms are all directly related to the chemical degradation of diester-diterpenoid alkaloids (e.g., aconitine and hypaconitine) into monoester-diterpenoid alkaloids (e.g., benzoylaconine and benzoylhypaconine). The degradation pathways include the cleavage of the C-8 ester bond of diester-diterpenoid alkaloids under high-temperature steam to form monoester-diterpenoid alkaloids, whose toxicity is 1/200 to 1/500 that of diester diterpenoid alkaloids. As hydrolysis conditions intensify, the C-14 ester bond breaks, forming amino alcohol-type aconitine, whose toxicity is 1/2000 to 1/4000 that of diester-type aconitine (HE, 2022): (1) Black shunpian: Mud aconite is washed and dipped in gall bladder water for a few days, boiled until it is heated through the heart, water-bleached and sliced, dipped and bleached to adjust the color, baked to half-dry after steaming, and then finally, dried in the sun or dried. After processing, the structure of biester-type alkaloids in aconite is destroyed and hydrolyzed into the low-toxicity mono-ester-type alkaloids. After processing by boiling for 8 min, water soaking and rinsing four times within 24 h, steaming for 3 h, and drying at 60 °C for 7.5 h, the hypaconitine and aconitine content in processed *Aconitum carmichaelii* slices fell below the detection limit. Additionally, the total content of diester-diterpenoid alkaloids significantly decreased after steaming, while the content of monoester-diterpenoid alkaloids notably increased, likely due to the conversion of diester-diterpenoid alkaloids during the steaming process (Ch, 2020; Gong et al., 2022). (2) Salt-processed aconite: Large and uniform aconite is washed, immersed in gall bladder water overnight and then soaked in salt and sun-dried daily until there is a large amount of salt cream on the surface and the texture becomes hard. This concocting process decreases the total alkaloid content in aconite to achieve the purpose of reducing the toxicity; however, the efficacy of aconite is also decreased after salt processing (Ch, 2020; Peng et al., 2022). (3) White sliced aconite: Uniform-sized mud aconites are soaked in brine for several days, boiled until thoroughly cooked, then peeled and longitudinally sliced. After water soaking, steaming, and sun-drying processes, HPLC testing showed that although the diester-type alkaloid content in the peeled aconite was reduced to approximately one-eighth the amount in raw aconite, it remained significantly higher than in other processed products. The white sliced aconite processing method involves removing the epidermis and high-temperature boiling, which decreases the diester-type alkaloid content and increases monoester-type alkaloid levels. Additionally, 5-hydroxymethylfurfural, a distinctive component not found in other processed forms, emerges, likely due to the high-temperature hydrolysis of *Vitis vinifera* sugars or fructose after epidermal removal (Zhu et al., 2011; Ch, 2020; Zhang C. et al., 2024). (4) Prepared aconite lateral root slices (with glycyrrhiza and black beans): Salt-processed aconite is soaked in clean water to remove salt, then boiled with *Glycyrrhiza uralensis* (common name: licorice root) and black beans until numbing sensations and bitterness disappear. *Glycyrrhiza uralensis* and the black beans are removed, then the aconite is sliced thinly and sun-dried. High-temperature steaming destroys the toxic components, while excipients help adsorb and promote the dissolution of toxic constituents. After processing with black beans and *Glycyrrhiza uralensis*, HPLC analysis shows increased monoester-diterpenoid alkaloid content and decreased diester-diterpenoid alkaloid levels (Guo et al., 2015; Ch, 2020; Yu et al., 2023). (5) Processed aconite lateral root slices: Black or white aconite lateral root slices are used as raw materials and sand-fried until puffed and slightly discolored. High-temperature processing reduces the diester-diterpenoid alkaloid content, thereby decreasing the leaching of toxic components. No diester-diterpenoid alkaloids (e.g., aconitine and neoaconitine) were detected in the processed slices. However, the monoester-diterpenoid alkaloid content decreased to 47.2, 58.1, and 67.1% of that in black aconite lateral root slices, respectively, with the total amount of the three monoester-diterpenoid alkaloids reduced to 54.9% of the raw product (Peng et al., 2019; Ch, 2020). The five detoxification mechanisms of processed aconite are summarized in Figure 2.”

Irrelevant plant terminology was used. For example, mentions of ‘*Broussonetia papyrifera*’ has been removed.

A correction has been made to the section 6 Overview of detoxification research, *6.2 Detoxification by compatibility*, 6.2.1 *Glycyrrhiza uralensis* combined with aconite, Paragraph number 1: “The combination of *Glycyrrhiza uralensis* and Aconite is commonly used in an herbal pair with classic formulations such as Gancao Fuzi Decoction and Sini Decoction. *Glycyrrhiza uralensis* contains triterpenoid saponins, like glycyrrhizic acid, and flavonoid active components, such as liquiritin, which mitigate the toxicity of aconite through various physical or chemical mechanisms (Wang X. X. et al., 2023; Dang et al., 2024). The common compatibility detoxification mechanisms are as follows: (1) Formation of complexes: Triterpenoid saponin components from *Glycyrrhiza uralensis* can reduce the content of diester-type alkaloids such as aconitine. The hydrolysis product, glucuronic acid from *Vitis vinifera*, combines with aconitine to form nontoxic complexes that are excreted through urine (Chen and Xu, 2006). The flavonoids in *Glycyrrhiza uralensis* (e.g., glycyrrhizin and liquiritin) can precipitate with the diester-diterpenoid alkaloids in aconite, reducing the leaching of toxic components and slowing the absorption of alkaloids in the intestines. After decocting *Glycyrrhiza uralensis* with aconite, the dissolution rate of *Glycyrrhiza uralensis* flavonoids decreases, likely due to the binding of hydroxyl groups in the flavonoids with the diester-diterpenoid alkaloids (Yang et al., 2003; Li Q. P. et al., 2018; Li W. et al., 2018). (2) Metabolic interference: The components of *Glycyrrhiza uralensis* induce the hepatic drug-metabolizing enzyme CYP3A4, elevate the expression levels of CYP3A4-related proteins, enhance enzymatic activity, and accelerate metabolism, thereby reducing peak plasma drug concentrations to achieve detoxification. When the cocktail probe drug method was employed to analyze the metabolism of aconitine in liver microsomes, the results demonstrated a significant acceleration in the metabolic rate of diester-type alkaloids, such as aconitine (Miao et al., 2014; Feng et al., 2024). (3) Regulation of ion channels: Aconitine exhibits significant cardiotoxicity by disrupting intracellular ion homeostasis, causing the sustained influx of Na^+^ and Ca^2+^. In contrast, the glycyrrhizin compounds in *Glycyrrhiza uralensis* can counteract the cardiotoxicity of aconite by acting on Na^+^ channels. For example, glycyrrhetinic acid at concentrations of 0.1, 1.0, and 10.0 μmol L-1 can act on L-type calcium channels in myocardial cells, inhibiting Ca^2+^ influx to exert detoxification effects (Xie et al., 2005). Moreover, when 2 mg/kg of glycyrrhiza uralensis flavonoids was administered to rats, they could antagonize the ventricular arrhythmia induced by aconitine (Ma et al., 2019).”

The original article has been updated.

